# The Effect of Food Allergen Exclusion on the Growth of Saudi Children

**DOI:** 10.3390/children10091468

**Published:** 2023-08-28

**Authors:** Manar Abdulaziz Bin Obaid, Sahar Abdulaziz AlSedairy, Hamza Ali Alghamdi, Ghzail M. Aljameel, Eman Alidrissi, Mofareh AlZahrani, Manal Abdulaziz Binobead

**Affiliations:** 1 Department of Food Science and Nutrition, College of Food and Agricultural Sciences, King Saud University, Riyadh 11451, Saudi Arabiagaljameel@hotmail.com (G.M.A.); mbinobead@ksu.edu.sa (M.A.B.); 2 Department of Pediatrics, King Fahad Medical City, Riyadh 12314, Saudi Arabia

**Keywords:** food allergy, milk, growth, fish, z-score, wheat, soybean

## Abstract

With a variety of symptoms that can impede children’s development, food allergies are an important public health concern. With the help of information from the King Fahad Medical City Hospital in Riyadh, we looked at how restricting certain foods affected the growth of Saudi children who had food allergies. An anonymous self-administered questionnaire asking about the individuals’ demographics and their restricted eating habits was completed by 72 children (48 boys and 24 girls) between the ages of 2 and 14. The sensitivity of six allergens (hen eggs, cow milk, fish, wheat, peanuts, and soybeans), anthropometric indices, specific Immunoglobulin E (IgE) levels, and sensitivity were examined. The Statistical Package for Social Science (SPSS), version 26, was used to analyze the data. Chi-square and *t*-tests were used to examine the relationships between various category variables. According to the findings, most of the mothers of the children were between the ages of 30 and 40 (80.6%), had a college degree (72.3%), were unemployed (59.7%), and had a monthly family income between 5000 and 15,000 SAR (69.4%). Both sexes had specific IgE antibodies for allergens in classes 2 and 3, with boys having noticeably (*p* ≤ 0.05) higher quantities than girls. While females were more sensitive to fish and peanuts, boys were more likely than girls to show specific IgE sensitivity to egg white, cow milk, wheat, and soybeans. Both sexes’ allergy levels were considerably (*p* ≤ 0.01) higher in children aged 5.01 to 10 than in other age groups. In terms of classifications of thinness, overweightness, and obesity, boys were slenderer than girls, and a greater percentage of boys than girls were overweight or obese. The exclusion of hen eggs, cow milk, wheat, and peanuts from the diet had a significant and detrimental effect on body mass index (BMI) and height-for-age ratio among children with impaired growth, in contrast to the demographic factors, which had a significant and favorable effect on the growth of other children. In conclusion, restrictions on food allergens impairs growth in Saudi children, particularly boys’ growth.

## 1. Introduction

Food allergies are becoming more common around the world, with a troubling 3–6% prevalence among children in developed countries [1]. A food allergy is caused by genetic factors, but environmental factors, which may also cause epigenetic changes, appear to be the cause of this rapid increase [1,2]. Large population-based observational studies have also identified several factors that may contribute to an increased risk of developing a food allergy, such as the delayed introduction of allergenic foods to an infant’s diet, early skin barrier impairment, low levels of vitamin D in infants, and altered or reduced microbial exposure [3,4]. According to Gonzalez-González et al. [5], milk, egg, soy, and wheat are the four most prevalent dietary allergies in children. The most prevalent foods that cause allergies differ by country. For example, cow milk, hen egg, and peanut allergies are the most common allergies in the United Kingdom and Australia, whereas cow milk, hen egg, and wheat allergies are the most common in Japan [6]. Furthermore, differences in the type and number of trigger foods have been observed among various ethnic groups residing in the same country [6]. Common food allergens in Saudi Arabia include peanuts, hen eggs, and cow milk (12.9%) [7].

Many interventions tried in pregnant or breastfeeding women and infants appear to have little to no benefit in preventing food allergies, including dietary avoidance of food allergens, vitamin supplements, fish oil, probiotics, prebiotics, and symbiotics; however, it should be noted that the evidence in many cases remains uncertain [8]. In a randomized trial, six allergenic foods—peanut, cooked egg, cow’s milk, sesame, whitefish, and wheat—were systematically introduced to 1303 infants either at 3 months (early introduction) or 6 months (standard introduction), and the primary outcome of that study was the development of a food allergy to one or more of the six foods between 1 year and 3 years of age [9]. A significant decrease in the prevalence of peanut and egg allergies was seen in the subset of the early introduction group that ingested at least 2 g of each food protein per week, despite the study’s limitations due to high rates of non-adherence to dietary guidelines [9]. Although these findings were not statistically significant, the early introduction group showed a 20% overall reduction in food allergy in the primary analysis [9].

According to a separate investigation, introducing gluten between the ages of 4 and 6 months was associated with a lower prevalence of celiac disease [10]. Early introduction of other allergens such as peanuts during the early ages was also negatively correlated with the prevalence of asthma and atopic dermatitis among children [11]. Delaying the introduction of some foods, such as oats (>5 months) and wheat (>6 months), was significantly related to an elevated risk of allergy sensitization to food and inhalant allergens, according to data from a Finnish birth cohort that included 994 kids [12]. According to one study, it is possible to lower the risk of peanut and hen egg allergies in children if such foods are introduced into infants’ weaning diets early and consumed regularly [13]. Since the earlier advice to delay the introduction of such foodstuffs did not diminish the prevalence of food allergies, postponing the introduction of allergenic food into a child’s diet has come under scrutiny [14]. As an alternative, early oral doses may result in tolerance, according to preclinical and clinical research [15,16]. The introduction of non-allergenic supplementary foods at the appropriate time is another dietary factor in preventing allergic disorders. However, there are currently no precise guidelines from the standpoint of allergy risk [14].

Although avoiding allergic foods seldom impacts the nutritional quality of the diet, many processed foods contain substances comparable to those found in eggs and soy, which further restricts the variety of meals available and may affect the diet’s nutritional quality (17). A developing child’s diet mostly consists of milk and wheat, but other sources with comparable nutrients should also be included [17]. Energy is provided by the macronutrients of protein, carbohydrates, and fats in one’s diet. Inadequate substitution can raise the risk of certain macronutrient shortages and result in insufficient energy intake [18]. Avoidance diets must be carefully planned to ensure that protein and fat requirements are satisfied because foods like milk, eggs, and soy are significant sources of protein and fat [17]. Poor growth and morbidities associated with protein deficiency can also result from protein-deficient diets [19].

The significance of food allergy education has been highlighted not only for patients and caregivers but also for communities and families [20]. In particular, improving parent knowledge regarding recognizing and managing acute reactions may help reduce their severity in the future, and parents’ understanding of the ingredients listed on food packages is crucial [21,22]. Parents and families should understand how to manage food allergies and have a clear action plan in the event of accidental exposure to reduce the stress of a food allergy diagnosis. For instance, Gomaa et al. [23] studied the knowledge and awareness of food allergies among mothers of children with allergies. They reported that mothers with children previously diagnosed with food allergies generally had poor knowledge and awareness [23]. Moreover, parents were found to be ill-prepared to handle exclusion diets and unable to appropriately administer epinephrine in emergencies [24,25]. This may lead to further problems, as children with food sensitivities may have insufficient nutrient intake and growth, which is most commonly observed in children with cow milk allergies [26].

Additionally, parents of children with food allergies have a lower health-related quality of life than the general population [27]. Thus, the current study was conducted to determine how eliminating specific food allergens may affect the growth of Saudi children with a history of allergies to selected foods.

## 2. Materials and Methods

### 2.1. Study Design and Participants

From the King Fahad Medical City Hospital database in Riyadh, the mothers of 72 allergic children (48 boys and 24 girls) aged 2–14 years responded to an anonymous self-administered questionnaire requesting information on demographics and the children’s restricted foods. Anthropometric indices and specific immunoglobulin E (IgE) levels against six allergens (hen eggs, cow milk, fish, wheat, peanuts, and soybeans) were determined at the hospital. The sample size was estimated based on previous studies conducted in several countries on growth indicators and influencing factors in children with food allergies [28,29]. Only children who had undergone total IgE assay and specific allergen detection tests were included in this study. Those without specific allergen testing results were excluded. 

### 2.2. Questionnaire

A structured questionnaire validated and approved by experts was used to determine the child’s family’s demographic information, such as mother’s and children’s ages, educational levels, occupations, and total family monthly income. Furthermore, the questionnaire asked about excluded foods in the child’s diet, and the child and their mother were supposed to answer yes or no when asked about specific foods excluded by focusing on the food allergen under investigation and related products. The information obtained was gathered and presented as a frequency and percentage for each item.

### 2.3. IgE Level Determination 

Patients with a history of significant skin, digestive, or respiratory reactions at the hospital after exposure to any potential food allergen were tested for IgE levels. Unicap 100 (Pharmacia AB Diagnostics, Uppsala, Sweden) was used to determine specific IgE levels. A child was considered sensitized to a specific food allergen if the amount of specific IgE antibody was greater than 0.7 KU/L, as previously described by Justicia et al. [30]. Following those authors, the test results were classified as specific IgE for food: 0.35–0.7, class 1; 0.71–3.50, class 2; 3.51–17.50, class 3; 17.51–50.0, class 4; 50.1–100, class 5; ≥100, higher values were classified as class 6.

### 2.4. Anthropometric Measurements 

All anthropometric data were measured in the clinic using Detecto Electronic. Body mass index (BMI) was calculated as the weight-to-height ratio (kg/m^2^). BMI- and height-for-age ratios were calculated by plotting the z-scores of Saudi children and adolescents on the Saudi National Growth Chart [31]. According to this chart, the BMI for age was categorized as follows: overweight (˃+1 SD), obesity (˃+2 SD), normal (˂−1 to ˂+1 SD), and thin (˂−2 SD). Height for age was classified as high (˃+1 SD), very high (˃+2 SD), medium (˂−1 to ˂+1 SD), low (<−2 SD > −3 SD), and very low (˂−3 SD). 

Furthermore, data for children with food allergies were analyzed and compared to the World Health Organization (WHO) growth criteria as follows: BMI for age was categorized as overweight (>+1 SD), obesity (>+2 SD), normal (<+1 SD to >−2 SD), thin (<−2 SD to >−3 SD), and severely thin (<−3 SD). Height for age was defined as high (>+1 SD), very high (>+2 SD), medium (<+1 SD to >−2 SD), low (<−2 SD to >−3 SD), and very low (<−3 SD). A low weight-for-height ratio indicated wasting, a low height-for-age ratio indicated stunting, and a low weight-for-age ratio indicated that a child was underweight. 

### 2.5. Statistical Analysis

Statistical Package for the Social Sciences (SPSS, version 26, Chicago, IL, USA) was used to analyze the data. Continuous variables were described as the mean and standard deviation, and categorical variables were described as frequency and percentage. An independent sample *t*-test was used to compare the meaning of variables between sexes, and a Chi-square test was used to compare categorical variables between the sexes. Spearman’s rank correlation coefficients and simple linear regression analysis were used to determine the relationship between demographic factors as independent variables and anthropometric indices as dependent variables. Statistical significance was defined at ≤0.05.

### 2.6. Ethical Considerations 

This study was approved by the multicenter Subcommittee on the Ethics of Human and Social Research at King Saud University (Ref No. KSU-HE-21-538). It was also approved by the Health Cluster of the Institutional Review Board Committee at King Fahad Medical City (Ref No. 22-11).

## 3. Results

### 3.1. Parents’ Demographic Characteristics and Allergy Knowledge 

Table 1 displays the frequency distribution of the family of children with food allergies (n = 72) according to their demographic data as well as the children’s age. According to the findings, the mothers’ ages in the study sample were divided into three categories, with the highest percentage (80.6%) between the ages of 30 and 40 years. The majority of mothers (72.3%) had a university education. By contrast, the proportion of mothers with an intermediate or lower-level education was the lowest 5.60%. The majority of mothers did not work (59.7%), and nearly two-thirds of the families earned between 5000 and 15,000 SAR (approximately 1332.44 and 4000 USD, respectively) per month. The majority of boys with recorded ages ranged from 5.01 to 10 years (43.75%), followed by those aged 2 to 5 years (37.5%). Also, the majority of girls with recorded ages ranged from 5.01 to 10 years (37.5%), followed by those of age from 2 to 5 years (33.33%).

### 3.2. Children’s IgE Values and Sensitivity According to Level and Age 

Table 2 shows the children’s mean levels of IgE antibodies to various food allergens. Based on IgE antibody levels against hen egg whites, boys (8.965 KU/L) had significantly (*p* ≤ 0.05) higher IgE levels than girls (3.408 KU/L), indicating the majority of boys had class 3 allergies, while the majority of girls had class 2 allergies. There was no significant difference in IgE levels against cow milk between boys and girls, and most cases were categorized as class 3. For fish, most were categorized as class 1. There were significant (*p* ≤ 0.05) differences between boys and girls in IgE levels against wheat and soybeans. The majority of boys and girls were categorized as having class 3 allergies, with boys having significantly (*p* ≤ 0.05) higher values than girls. For the allergens under investigation, none of the children had IgE levels in classes 4, 5, or 6.

Table 3 depicts the frequency distribution of children with IgE levels categorized by food allergens (boys n = 48, girls n = 24). The majority of boys (35.42%) had moderate levels, with negative results (27.08%) against egg whites, while the majority of girls (29.17%) had negative levels, with low or medium levels (25%). Boys had extremely high levels, but none of the girls did. The majority of boys (29.17%) had moderate milk allergenicity, followed by those with negative results (25.0%), while the majority of girls (45.83%) had negative levels, followed by those with moderate levels (20.83%). Extremely high levels were found in a small percentage of boys and girls. The findings revealed that 89.58% of boys and 87.5% of girls had a fish allergy. However, 4.18% of boys and 12.5% of girls were allergic to fish, with one boy having an ultra-high allergy. The majority of boys (35.42%) and girls (33.32%) had a moderate allergy to wheat, with both having a high level. Wheat allergenicity was extremely high in boys (4.16%), with one person in each group having an ultra-high allergy. Peanut allergenicity was more prevalent in girls (45.83%) than in boys (31.25%), with a moderate level in both genders. It was discovered that 29.17% of both boys and girls had a high allergy to soybeans, but the percentage of girls (41.66%) with a moderate level outnumbered boys (25%). Boys (10.42%) were found to have a higher percentage of ultra-high soybean allergies than girls (4.17%), with only one boy suffering from extremely high levels.

Table 4 shows the frequency distribution of children (boys n = 48, girls n = 24) with IgE against allergens categorized according to age. According to the results, a significant (*p* ≤ 0.001) variation was observed between boys’ and girls’ ages and allergenicity against food allergens. The findings revealed that most boys aged 5.01–10 years (35.42%) were allergic to hen egg whites, followed by those aged 2–5 years (29.17%). However, the majority of girls (29.17%) aged 2–5 and 5.01–10 years tested positive for hen egg white allergens. In terms of milk allergen, a high percentage of boys aged 5.01–10 years (33.33%) were allergic to cow milk, followed by those aged 2–5 years (29.17%), while the majority of girls aged 2–5 years (25%) were allergic to cow milk, followed by those aged 5.01–10 years (20.83%). The majority of boys and girls were not allergic to fish with only a few cases of fish allergies. The findings revealed that a high percentage of boys (31.25%) aged 2–5 years and girls (33.33%) aged 5.01–10 years were suffering from wheat allergy. A high percentage of boys (39.58%) of the same age (5.01–10 years) were found to be allergic to peanuts and soybeans, while the highest percentage of girls aged 5.01–10 years were found to be allergic to peanuts and soybeans (37.5% and 33.33%, respectively).

### 3.3. Children’s Food Exclusion

Table 5 shows the types of foods excluded by children under investigation. According to the questionnaire, there were significant (*p* ≤ 0.05 or *p* ≤ 0.01) differences between excluded and included foods for both genders. The results showed that more than half of the boys were excluded from taking egg white (54.2%), milk (60.4%), and peanuts (75%), while those for the girls included egg white (66.7%), milk (50%), peanut (83.3%), and soybean (50%). The second excluded foods for boys included wheat (47.9%), sesame (47.9%), pistachio (37.5%), soybean (37.5%), fish (29.2%), banana, and hazelnut (27%), while those for girls included wheat (37.9%), sesame (37.5%), pistachio and hazelnut (33.3%), cashew (29.2%), and fish (25%). Other foods were excluded, but only with a small percentage of respondents. 

### 3.4. BMI- and Height-for-Age of Children

Table 6 displays the BMI-for-age and height-for-age measurements of children with food allergies based on the WHO classification and the National Growth Charts for Saudi Arabia. According to the WHO classification, a high percentage of both boys and girls were of average weight (58.32% and 58.33%, for boys and girls, respectively), followed by those who were overweight, which was more common in boys (20.83%) than in girls (16.67%). In addition, boys (16.67%) had a higher percentage of obesity than girls (12.50%) in the study sample. Girls (8.34%) were found to be thinner than boys (4.17%). According to the Chi-square test, there was a significant difference in BMI-for-age (*p* = 0.05) between boys and girls. Furthermore, the majority of children (62.50% of girls and 52.08% of boys) had normal height-for-age ratios. Compared with girls (4.17%), boys had a higher height-for-age ratio (29.17%). However, 20.83% of the girls had a higher ratio than the boys (4.17%). Boys suffered from severe stunting at a rate of 8.33%, whereas girls did not. According to the Chi-square test, there was a significant difference in the height-for-age ratio between boys and girls (*p* = 0.032). 

According to the National Growth Charts for Saudi Arabia, there was a significant difference in BMI-for-age classification degree between the sexes (Chi-square = 0.011). The classification revealed that the majority of the boys (56.25%) and girls (50%) had normal BMI-for-age ratios. However, girls were more overweight (25.00%) than boys (12.50%), while boys were thinner (16.67%) than girls (12.50%). Additionally, boys had a higher obesity rate (14.58%) than girls (12.50%). The results revealed a significant difference in the degree of height-for-age index classification between the sexes (Chi-square = 0.021 *). More than half of the children (boys, 56.25%; girls, 54.17%) had an average ratio; however, the height-for-age ratio was higher in boys (27.08%) than in girls (12.5%). Approximately 8.34% of boys suffered from severe stunting. When we compared the BMI-for-age and height-for-age ratios for children with food allergies using the National Growth Charts for Saudi Arabia and the WHO classification, children were thinner in the Saudi national reference than in the WHO reference (15.28 vs. 8.34%). Although the prevalence of overweightness in Saudi children with food allergies was lower than the national average, the difference was not statistically significant (18.06% vs. 16.67%). Both references had the same median height-for-age (55.56%). Finally, the Saudi national reference reduced short stunting to a greater extent than the WHO reference (4.16% vs. 8.33%). Subsequently, the national reference increased to a high ratio (12.50% vs. 9.72%).

### 3.5. Factors Associated with Children’s Growth

Table 7 depicts the correlation between the allergen type, as well as demographic factors (child’s age and mother’s education, income, and work) (independent variables) and other child’s anthropometric indices (BMI and height-for-age) (dependent variables). All tested types of food allergens including egg white, cow milk, peanuts, wheat, and chocolate were found strongly but negatively associated (*p* < 0.05) with respondents’ anthropometric indices. However, no association between fish and soybeans with the BMI and height-for-age was seen among all tested children (Table 7). On the other hand, a positive correlation was seen between these two anthropometric indices when tested against children’s age, the age of the child at the time of diagnosis, and mother’s factors including education, income, and work (*p* ≤ 0.01).

## 4. Discussion

The major finding of this study shows that restrictions on food allergen foods restrict children’s, especially boys, growth in Saudi Arabia. In accordance, the determination of the IgE for the respondents in this study showed that the majority of boys and girls were categorized as having class 3 allergies, with boys having significantly (*p* ≤ 0.05) higher values than girls. Higher levels of specific IgE were observed in egg whites, cow milk, wheat, peanuts, and soybeans for boys, while for girls this was the case for cow milk and peanuts. Specific IgE against allergens is a defining feature of allergic disease [32]. Therefore, the results revealed that both boys and girls had allergic symptoms, as indicated by the values obtained for IgE in their serum. According to Du Toit et al. [33], the most well-known types of food hypersensitivity disorders are IgE-mediated reactions, which are also responsible for many allergy symptoms.

Moreover, to determine whether a patient has IgE antibodies to specific foods, skin prick tests and/or various in vitro tests (e.g., RAST) are used [34]. Previous research, however, has found that patients with food specific IgE antibodies experience clinical symptoms when the food is consumed [35]. Also, previous research on children with atopic dermatitis and allergies to cow’s milk, eggs, or peanuts found that high levels of allergen-specific IgE antibodies were predictive of food-induced clinical symptoms [36]. In a study of food allergies in Swiss children using skin prick tests and the detection of specific IgE levels in sera, hen egg allergy was detected in 23.7% of the patients, which was the highest percentage among the studied allergies [37]. Al-Ghonaim et al. [38] discovered a significant increase in total and specific IgE levels in four wheat allergy patients. Cow milk and dairy products, hen eggs, peanuts, nuts, gluten-containing cereals (e.g., wheat, rye, barley), sesame, soybeans, mustard, fish, crustaceans, and shellfish have been identified as the most allergenic foods [39].

The frequency distribution of children according to specific IgE sensitization against food allergens revealed that boys were more likely than girls to be more sensitive to egg white, cow milk, wheat, and soybeans, while girls were more sensitive to fish and peanuts. The results indicated that the sensitivity against food allergens differed between boys and girls, with higher percentages of allergic boys than girls. Most studies show that males have a higher prevalence of IgE-sensitization than females, at least until adolescence [40,41]. Regarding specific IgE sensitization, females and males differ [42]. However, Chen et al. [43] reported that the prevalence of allergic diseases varies between genders and can be higher in either gender depending on age and disease, which cannot be explained solely by differences in IgE-sensitization. Salo et al. [44] compared specific IgE levels for 19 specific IgEs concerning sex in participants aged 6 and up and observed that only two specific IgEs differed by sex against milk and Aspergillus fumigatus allergens. Male sex is a risk factor for early sensitization to food and aeroallergens [45], hypersensitivity reactions to food, and food-induced anaphylaxis in childhood [46]. This may be explained by sex differences in immune response profiles during childhood [47]. One possible explanation for the gender–food allergen relationship is that estrogens boost humoral immunity and antibody synthesis, while androgens and progesterone suppress immunity and inflammation [43]. Increased estrogen in girls with age may increase the prevalence of food allergies with age [43].

The frequency distribution of children with specific IgE against food allergens classified by age (years) revealed that boys and girls aged 5 to 10 years were more allergic regardless of allergen type. In both sexes, the percentages of allergic respondents decreased significantly with age. This can be explained by the fact that the majority of children developed a tolerance to these common allergens with age, as reported by Sicherer and Sampson [48]. Furthermore, the median age at food–allergy onset was two years earlier in boys than in girls (5 years). Other studies have shown that, after puberty, female patients outnumber male patients in terms of IgE-mediated food allergies and hospitalizations for food-induced anaphylaxis [46]. Kumata et al. [49] discovered a link between IgE antibody concentrations in wheat and interaction with wheat. Also, age affected the outcome, with younger children having a stronger link between serum-specific IgE concentrations and challenge outcomes than older children. Shek et al. [50] studied food-specific IgE levels in cow’s milk and hen’s egg allergies over time. They discovered a link between the degree of decrease in food-specific IgE antibody concentrations over time and the likelihood of developing tolerance.

Some children take certain types of milk while breastfeeding, and when the breastfeeding period ends, the children gradually begin to take food. Some of these children develop allergies while breastfeeding, while others develop allergies after the breastfeeding period ends. The developed allergies force their families to exclude foods associated with those allergies. The main foods that are excluded are egg white, cow milk, peanuts, wheat, soybeans, and a few others. Although avoiding allergic foods has little effect on nutritional quality, many processed foods contain substances similar to those found in eggs and soy, further limiting the variety of meals available [17]. A growing child’s diet consists primarily of milk and wheat, but other sources of comparable nutrients should also be included [17]. The macronutrients protein, carbohydrates, and fats in one’s diet provide energy. Inadequate substitution can increase the risk of certain macronutrient shortages and insufficient energy intake [51]. Because foods like milk, eggs, and soy are high in protein and fat, they must be carefully planned for a child’s diet to ensure that protein and fat requirements are met. A separate study [47] found that introducing gluten between the ages of 4 and 6 months was associated with a lower prevalence of celiac disease. Moreover, according to data from a Finnish birth cohort of 994 children, delaying the introduction of some foods, such as oats (>5 months) and wheat (>6 months), was significantly associated with an increased risk of allergy sensitization to food and inhalant allergens [21]. In contrast, asthma and atopic dermatitis were two other allergy disorders discovered to be allergen-specific and unaffected by the early introduction of peanuts [26].

Despite removing some foods from the children’s diet, with most removing some foods from the children’s diet, most boys and girls had normal BMI-for-age and height-for-age ratios based on WHO reference values and Saudi national growth reference values. There was a significant difference between boys and girls in terms of thin, overweight, and obese; boys were thinner than girls, while the number of overweight or obese boys outnumbered that of girls. The majority of studies on the relationship between food allergies and children’s development have found that food sensitivity is a risk factor for developmental impairment due to the elimination of diets associated with food allergies. [52,53]. Visness et al. [54] discovered a link between obesity and atopy in American children aged 2 to 19. Furthermore, they reported that the associations for most of the outcomes were stronger for the obese category than for the overweight category, indicating a dose-response for weight, and the analysis of continuous BMI with total IgE levels supports the concept that increased weight is associated with increased allergic general tendency. Total IgE levels were higher in girls than in boys in the overweight category but higher in boys than in girls in the obese category, and the relationship between BMI and total IgE levels was stronger in girls than in boys [54]. Hayashi et al. [55] investigated the relationship between obesity and the prevalence of food allergies and discovered a positive relationship between obesity and the prevalence of food allergies in girls. A previous study in the United States that looked at the relationship between obesity, serum IgE, and allergic symptoms concluded that obesity might be a factor in the increased prevalence of allergic disease in children, particularly food allergies [54]. In contrast, a study of Vietnamese children found no significant link between obesity and food allergies [56].

Spearman correlation and simple regression analysis were used to determine the factors associated with children with impaired growth. The results showed that the absence of hen eggs, cow milk, wheat, and peanuts from the diet significantly negatively impacted BMI-for-age and height-for-age ratios in both boys and girls, with cow milk having a stronger association than other allergens. According to ref. [41], children with food restrictions have lower calories, proteins, carbohydrates, fats, riboflavin, vitamin B12, phosphorus, calcium, and iron intake, which hampers their growth. Allergy severity was an independent risk factor for height and weight. The children with food restrictions had significantly lower height, weight, head circumference, and mid-upper arm circumference than those in the non-food-restricted group. More stunted and underweight children were found in the food-restricted group [41]. Despite the negative effects of food allergen exclusion on child growth, it appears that the effect is limited to children with lower BMI and height-to-age ratios. We anticipate that the children did not receive any food to compensate for the nutrient shortage.

This study emphasizes the importance of intensive food allergy health education programs for managing food allergies and potential treatments for mothers whose children have suffered from growth impairment. On the other hand, child age, age at diagnosis, mother’s education, and family monthly income had a significant positive impact on BMI-for-age and height-for-age ratios in normal, overweight, and obese boys and girls. The authors propose that boys and girls with normal growth, overweightness, and obesity received food to compensate for the nutrient shortage as indicated by the demographic characteristics that favor their nutritional status. A well-educated mother, reasonable family income, and the mother’s presence at home throughout the day, as well as the child’s age, all contributed to the children eating a balanced diet. Internal factors, such as the child’s taste and food preferences, as well as external factors, such as peers, media, and parents, influence eating habits. Parents play an important role in early childhood because they serve as providers, enforcers, and role models for children who are still very dependent on them [57]. Some of these efforts succeed in meeting the parent’s objectives. For example, feeding practices such as making healthy foods available and modeling healthy food consumption have been shown to facilitate healthy eating behaviors in young children [58]. Furthermore, a recent prospective observational study found that tracking a child’s intake of high energy-dense foods at the age of two was associated with a healthier weight status one year later [59]. Kröller and Warschburger [60] discovered that mothers with higher education used more monitoring of their children’s food intake.

This study’s limitations include the: Food allergy patients were located through the King Fahad Medical City Hospital database in Riyadh, and the children under investigation may not have been completely representative of the local population. Furthermore, a significant number of children were recruited directly through hospital emergency departments. Therefore, it can be speculated that the children included in this study represented a subset of infants and young children with more severe food allergies. Furthermore, it should be noted that the purpose of this study was not to determine the prevalence of food allergies in Saudi children but to examine the clinical manifestations and distribution of allergens that are most commonly involved in cases confirmed with food allergies.

## 5. Conclusions

As indicated by specific IgE antibody levels and sensitivity, both boys and girls were allergic to one or more of the allergens tested. Our findings suggest that food allergen restriction has a negative correlation with the growth of some children, particularly boys. Although most of the mothers in this study were young, educated, unemployed, and had a decent monthly income, some children faced growth impairment. Therefore, a food allergy health education program should be developed and implemented to educate parents and caregivers about food allergies and treatment options.

## Figures and Tables

**Table 1:** Demographic and social characteristics of the mothers of participating children and children’s ages (n = 72).

Demographic Character	No.	%		
Mothers’ Age				
<30 years	3	4.2		
30–40 years	58	80.6		
>40 years	11	15.3		
Mothers’ Education				
Primary school or less	4	5.60		
Secondary school	16	22.2		
University or above	52	72.3		
Mothers’ Work				
Housewife	43	59.7		
Education sector	17	23.6		
Health field	7	9.70		
Other	5	6.90		
Family’s monthly Income (SR)				
<5000	5	6.90		
5000–15,000	50	69.4		
16,000–25,000	13	18.10		
≥25,000	4	5.60		
Children’s age (year)	Boys		Girls	
	No.	%	No.	%
2–5	18	37.5	8	33.33
5.01–10	21	43.75	9	37.5
10.01–14	9	18.75	7	29.17
Total	48	100	24	100

**Table 2:** Average specific IgE levels for boys (n = 48) and girls (n = 24) with food allergies according to gender according to *t*-test.

Foodallergen IgE (KU/L)	Gender		*t*-Test	*p*-Value
	Boys (n = 48)	Girls (n = 24)		
	Mean ± SD	Mean ± SD		
Egg white	8.965 ± 4.27	3.408 ± 1.54	1.020 *	0.050
Cow milk	8.914 ± 4.16	9.932 ± 4.90	−0.264	0.793
Fish (cod)	1.632 ± 0.936	1.01 ± 0.832	0.490	0.611
Wheat	10.853 ± 4.57	6.588 ± 2.46	0.843 *	0.042
Peanut	16.589 ± 8.05	14.370 ± 5.02	0.351	0.849
Soybean	12.570 ± 4.47	7.01 ± 2.48	1.296 *	0.038

* *p* ≤ 0.05; KU/L = kilounits per lite. Specific IgE for food: 0.35–0.7, class 1; 0.71–3.50, class 2; 3.51–17.50, class 3; 17.51–50.0, class 4; 50.1–100, class 5; ≥100; higher values were classified as class 6.

**Table 3:** Levels of sensitization against food allergens in participating children.

Allergen/Level	Gender				Total	
	Boys (n = 48)		Girls (n = 24)			
	No.	%	No.	%	No.	%
Egg white						
Negative	13	27.08	7	29.17	20	27.78
Low	5	10.42	6	25.00	11	15.28
Moderate	17	35.42	6	25.00	23	31.94
High	9	18.75	4	16.67	13	18.06
Very high	1	2.08	1	4.16	2	2.77
Extremely high	3	6.25	-	-	3	4.17
Cow milk						
Negative	12	25.0	11	45.83	23	31.94
Low	10	20.83	3	12.50	13	18.05
Moderate	14	29.17	5	20.83	19	26.39
High	8	16.67	1	4.17	9	12.50
Very high	1	2.08	2	8.33	3	4.17
Ultra-high	1	2.08	1	4.17	2	2.78
Extremely high	2	4.17	1	4.17	3	4.17
Fish						
Negative	43	89.58	21	87.50	64	88.88
Low	1	2.08	-	-	1	1.39
Moderate	1	2.08	-	-	1	1.39
High	2	4.18	3	12.50	5	6.95
Ultra-high	1	2.08	-	-	1	1.39
Wheat						
Negative	6	12.50	7	29.17	13	18.06
Low	6	12.50	1	4.17	7	9.72
Moderate	17	35.42	8	33.32	25	34.72
High	11	22.92	7	29.17	18	25.0
Very high	5	10.42	-	-	5	6.94
Ultra-high	1	2.08	1	4.17	2	2.78
Extremely high	2	4.16	-	-	2	2.78
Peanut						
Negative	6	12.50	1	4.17	7	9.72
Low	4	8.33	2	8.33	6	8.33
Moderate	11	22.92	5	20.83	16	22.22
High	15	31.25	11	45.83	26	36.11
Very high	7	14.58	3	12.50	10	13.89
Ultra-high	2	4.17	1	4.17	3	4.17
Extremely high	3	6.25	1	4.17	4	5.56
Soybean						
Negative	6	12.50	4	16.67	10	13.89
Low	9	18.75	1	4.17	10	13.89
Moderate	12	25.00	10	41.66	22	30.56
High	14	29.17	7	29.16	21	29.16
Very high	1	2.08	1	4.17	2	2.78
Ultra-high	5	10.42	1	4.17	6	8.33
Extremely high	1	2.08	-	-	1	1.39

No. = Number.

**Table 4:** Frequency distribution of children with specific IgE sensitivity between boys and girls against food allergens categorized according to age (years).

Allergen/Age (Year)	Boys (n = 48)				*p*-Value	Girls (n = 24)				*p*-Value
	Negative		Positive			Negative		Positive		
	No.	%	No.	%		No.	%	No.	%	
Egg white (KU/L)										
2–5	4	8.33	14	29.17	<0.001 **	1	4.17	7	29.17	<0.001 **
5.01–10	4	8.33	17	35.42		2	8.33	7	29.17	
10.01–14	5	10.42	4	8.33		4	16.67	3	12.5	
Cow milk (KU/L)										
2–5	4	8.33	14	29.17	<0.001 **	2	8.33	6	25	<0.001 **
5.01–10	5	10.42	16	33.33		4	16.67	5	20.83	
10.01–14	3	6.25	6	12.5		4	16.67	3	12.5	
Fish (KU/L)										
2–5	14	29.17	4	8.33	<0.001 **	8	33.33	0	0	<0.001 **
5.01–10	20	41.67	1	2.08		7	29.17	2	8.33	
10.01–14	9	18.75	0	0		6	25	1	4.17	
Wheat (KU/L)										
2–5	3	6.25	15	31.25	<0.001 **	4	16.67	4	16.67	<0.001 **
5.01–10	20	41.67	1	2.08		1	4.17	8	33.33	
10.01–14	1	2.08	8	16.67		2	8.33	5	20.83	
Peanut (KU/L)										
2–5	3	6.25	15	31.25	<0.001 **	7	29.17	1	4.17	<0.001 **
5.01–10	2	4.17	19	39.58		0	0	9	37.5	
10.01–14	1	2.08	8	16.67		1	4.17	6	25	
Soybean (KU/L)										
2–5	3	6.25	15	31.25	<0.001 **	7	29.17	1	4.17	<0.001 **
5.01–10	2	4.17	19	39.58		1	4.17	8	33.33	
10.01–14	1	2.08	8	16.67		2	8.33	5	20.83	

** *p* ≤ 0.01/Independent samples *t*-test. F = frequency.

**Table 5:** Frequency distribution of participants according to excluded and unexcluded foods.

Excluded Foods	Boys (n = 48)				Chi-Square	Girls (n = 24)				Chi-Square
	Excluded		Unexcluded			Excluded		Unexcluded		
	No.	%	No.	%		No.	%	No.	%	%
Eggs white	26	54.2	22	45.8	0.564	16	66.7	8	33.3	0.011 *
Cow milk	29	60.4	19	39.6	0.049 *	12	50.0	12	50.0	1.00
Fish	14	29.2	34	70.8	0.004 **	6	25.0	18	75.0	0.014 *
Tuna	5	10.4	43	89.6	0.001 **	0	0.0	24	100.0	0.001 **
Banana	13	27.1	35	72.9	0.001 **	5	20.8	19	79.2	0.004 **
Peanuts	36	75.0	12	25.0	0.001 **	20	83.3	4	16.7	0.001 **
Hazelnut	13	27.1	35	72.9	0.001 **	8	33.3	16	66.7	0.011 *
Almond	11	22.9	37	77.1	0.005 **	5	20.8	19	79.2	0.004 **
Chocolate	0	0.0	48	100.0	0.001 **	3	12.5	21	87.5	0.001 **
Strawberry	5	10.4	43	89.6	0.001 **	3	12.5	21	87.5	0.001 **
Shrimp	8	16.7	40	83.3	0.001 **	2	8.3	22	91.7	0.001 **
Wheat	23	47.9	25	52.1	0.773	9	37.5	15	62.5	0.021 *
Soybean	18	37.5	30	62.5	0.083	12	50.0	12	50.0	1.00
Oyster	0	0.0	48	100.0	0.001 **	0	0	24	100.0	0.001 **
Sesame	23	47.9	25	52.1	0.773	9	37.5	15	62.5	0.021 *
Pistachio	18	37.5	30	62.5	0.083	8	33.3	16	66.7	0.011 *
Peans	9	18.8	39	81.3	0.001 **	5	20.8	19	79.2	0.004 **
Cashew	7	14.6	41	85.4	0.001 **	7	29.2	17	70.8	0.041 *
Kiwi	5	10.4	43	89.6	0.001 **	2	8.3	22	91.7	0.001 **
Tomatoes	3	6.3	45	93.8	0.001 **	2	8.3	22	91.7	0.001 **
Flavor	4	8.3	44	91.7	0.001 **	1	4.2	23	95.8	0.001 **

* *p* ≤ 0.05; ** *p* ≤ 0.01, F = Frequency.

**Table 6:** BMI-for-age and height-for-age of the children with food allergies according to WHO and Saudi national growth chart z-score standard deviation (SD).

Nutritional Index	WHO Reference				National Growth Reference			
	Boys		Girls		Boys		Girls	
	No.	%	No.	%	No.	%	No.	%
BMI-for-age								
Severe thinness	2	4.17	-	-	-	-	-	-
Thinness	2	4.17	2	8.34	8	16.67	3	12.50
Normal	28	58.32	14	58.33	27	56.25	12	50.00
Overweight	8	16.67	5	20.83	6	12.50	6	25.00
Obesity	8	16.67	3	12.50	7	14.58	3	12.50
Total	48	100.0	24	100.0	48	100.0	24	100.0
Height-for-age								
Very low	4	8.33	-	-	4	8.34	-	-
Low	3	6.25	3	12.50	1	2.08	2	8.33
Medium	25	52.08	15	62.50	27	56.25	13	54.17
High	14	29.17	1	4.17	13	27.08	3	12.5
Very High	2	4.17	5	20.83	3	6.25	6	25.0
Total	48	100.0	24	100.0	48	100.0	24	100.0

Saudi National Growth Chart z-score, Chi-square = 0.021; WHO scale, Chi-square = 0.032.

**Table 7:** Children’s (n = 72) anthropometric indices.

Dependent Variables/ Independent Variables	Height-for-Age		*p*-Value	BMI-for-Age		*p*-Value
	r	(β, r^2^)		r	(β, r^2^)	
Egg white	−0.279 *	(−0.045 *, 0.077)	0.050	−0.184 *	(−0.021 *, 00.034)	0.013
Cow milk	−0.317 **	(−0.008 **, 0.100)	0.008	−0.223 *	(−0.131 *, 0.054)	0.027
Fish	−0.177	(−0.001, 0.031)	0.869	−0.134	(−0.027, 0.017)	0.821
Peanuts	−0.183 *	(−0.029 *, 0.033)	0.050	−0.204 *	(−0.019 *, 0.041)	0.017
Wheat	−0.139 *	(−0.005 *, 0.019)	0.044	−0.042 *	(−0.004 *, 0.002)	0.043
Soybean	−0.156	(−0.040, 0.024)	0.304	−0.037	(−0.003, 0.001)	0.789
Chocolate	−0.114 *	(−0.110 *, 0.013)	0.041	−0.072 *	(−0.087 *, 0.005)	0.041
Child age (years)	0.836 **	(0.201 **, 0.698)	0.004	0.436 **	(0.344 **, 0.190)	0.003
Age at diagnosis	0.495 **	(0.551 **, 0.245)	0.001	0.339 **	(0.044 **, 0.115)	0.004
Mother Education	0.495 **	(0.551 **, 0.245)	0.001	0.339 **	(0.044 **, 0.115)	0.004
Monthly Income	0.095 *	(0.107 *, 0.009)	0.011	0.125 **	(0.017 *, 0.014)	0.014
Mother work	−0.118	(−0.015, 0.044)	0.323	−0.096	(−0.011, 0.017)	0.423

* *p* ≤ 0.05; ** *p* ≤ 0.01; r = correlation coefficient; β = regression coefficient; partial r^2^ for independent variables of interest.

## Data Availability

The datasets used and analyzed during the current study are available from the corresponding author upon reasonable request.
